# Completion Colonoscopy: Should We Select the Endoscopist Based on Which Noninvasive Colorectal Cancer Screening Test was Positive?

**DOI:** 10.14309/ctg.0000000000001000

**Published:** 2026-03-12

**Authors:** Brian C. Jacobson

**Affiliations:** 1 Gastroenterology Division, Mass General Brigham and Harvard Medical School, Boston, Massachusetts, USA.

With increasing reliance on stool and blood-based colorectal cancer screening, there will be a commensurate increase in the number of requests for completion colonoscopies, i.e. colonoscopies performed to investigate positive noninvasive testing (1). Traditionally, patients referred for screening colonoscopy are distributed among all endoscopists in a practice, regardless of the endoscopists' comfort level with performing resection of larger, advanced neoplasms. The reason this process works is the relatively low rate of finding a large, advanced neoplasm among average risk individuals undergoing colorectal cancer screening (2). Data through June 2023 from the New Hampshire Colonoscopy Registry, for example, indicate the presence of an adenoma ≥20 mm in 1,382 (0.8%) patients among 162,894 screening and surveillance colonoscopies (Joseph C. Anderson, personal communication). However, when an endoscopist encounters a larger polyp they are not comfortable resecting, typically because of lack of training or experience, they refer these patients for a repeat colonoscopy with a more advanced endoscopist for complex polypectomy, endoscopic mucosal resection, or endoscopic submucosal dissection.

This 2-stage colonoscopy practice exists because of an unspoken acceptance that a small percentage of patients must undergo 2 procedures. In some ways, we already accept a relatively high repeat screening colonoscopy rate based on inadequate bowel preparation. Current guidance from both the US Multi-Society Task Force on Colorectal Cancer and the European Society of Gastrointestinal Endoscopy calls for a bowel preparation adequacy rate of ≥90% (3,4), meaning that we are willing to accept up to 10% of patients needing to undergo 2 colonoscopies to complete screening.

However, in a truly patient-centered approach, one might consider restricting the performance of some completion colonoscopies to those physicians who perform complex polypectomies, expressly to eliminate the need for a second colonoscopy if an advanced neoplasm is diagnosed. This is particularly relevant for those patients who chose a noninvasive colorectal cancer screening test *specifically* to avoid colonoscopy in the first place (5).

This change in approach to scheduling, referring average risk screening patients to all endoscopists but referring patients in need of a completion colonoscopy only to more advanced endoscopists, would make sense only if the likelihood of finding a large, advanced neoplasm exceeds some acceptable rate. For example, if the likelihood is comparable to finding such polyps during an average risk screening, then there is no need for directed scheduling. However, because noninvasive colorectal cancer screening tests are becoming more prevalent and their performance characteristics for predicting advanced neoplasia increase, there may come a time to incorporate a new approach.

Consider the *Summary of Safety and Effectiveness Data* for Shield (6), Cologuard (7), and Cologuard Plus (8) available from the Food and Drug Administration (see Table 1).

**Table 1.** Summary of safety and effectiveness data from the Food and Drug Administration (6–8)

	Number of subjects	Number of positive tests	Total % of positive tests with polyps ≥20 mm	% Of positive tests with polyps 20–29 mm	% Of positive tests with polyps ≥30 mm
Shield^TM^	7,861	899	4	2	2
Cologuard^TM^	10,023	1,613	5	3	2
Cologuard Plus^TM^	18,911	2,497	9	6	3

Based on these noninvasive colon cancer screening tests' landmark studies, the percentage of subjects with a positive Shield, Cologuard, and Cologuard Plus who had an adenoma 20 mm or larger was 4%, 5%, and 9%. Many endoscopists are not comfortable resecting lesions ≥20 mm, and the US Multi-Society Task Force on Colorectal Cancer specifically recommends that an endoscopist experienced in advanced polypectomy manage large (≥20 mm) nonpedunculated colorectal lesions (9). This has implications for the informed consent process: If you knew you had a 5%–9% chance, based on a noninvasive test, that your endoscopist would complete your colonoscopy and then send you to a colleague for a second colonoscopy, would you prefer to have your first colonoscopy with that colleague instead?

Those who schedule colonoscopies could assign patients to endoscopists specifically based on which noninvasive test was positive based on the test's performance characteristics. Certain patients might therefore be scheduled only with those endoscopists who profess comfort with resection of large polyps. In some practices, this may be a large percentage of endoscopists, but in others, there may be only a few.

This highlights what is typically missing in the publicly available data from analyses of noninvasive tests; namely, the frequency of adenomas that required a second colonoscopy for resection. Typically reported is the frequency of advanced adenomas, a composite that can include both smaller villous or sessile serrated lesions, making it difficult to determine the likelihood that a more advanced endoscopist would be the better choice for the completion colonoscopy. This calls for the inclusion of more detailed performance characteristics as new tests emerge so that we can better direct our patients to the most appropriate endoscopists and thereby decrease the rate of repeat colonoscopies. This may be particularly helpful for future tests if they have low sensitivity for small adenomas but higher sensitivity for larger, nonmalignant lesions.

One implication with this approach is that it behooves endoscopists to learn the techniques of complex polypectomy, endoscopic mucosal resection, and perhaps even hybrid endoscopic submucosal dissection, to decrease the need for repeat colonoscopy just to resect a lesion. As we enter this new era of burgeoning 2-stage colorectal cancer screening, and greater attention to patient-centered care, the American College of Gastroenterology and other societies might consider increasing educational efforts to improve endoscopists' comfort with complex polypectomy.

In summary, as we rely on more 2-stage colorectal cancer screening, using a stool- or blood-based test followed by completion colonoscopy when those tests are positive, we should consider the likelihood the endoscopist will be required to resect a large lesion. Personalized referrals for colonoscopy by more advanced endoscopists could be made based on each noninvasive test's predictive properties, which should be reported in more detail in published studies to include how often more complex polypectomies were required during pivotal clinical trials. A better long-term strategy is to have more endoscopists trained to perform complex polypectomy, thereby improving their comfort with such procedures and limiting the need for a second completion colonoscopy.

## CONFLICTS OF INTEREST

**Guarantor of the article:** Brian C. Jacobson, MD, MPH, FACG.

**Specific author contributions:** Ideation and writing by Brian C. Jacobson.

**Financial support:** None to report.

**Potential competing interests:** None to report.

## References

[1] Hosseini-Carroll P Dominitz JA Jacobson BC American Society for Gastrointestinal endoscopy colorectal cancer screening project national summit. Gastrointest Endosc 2025;101(3):511–9. 40024634 10.1016/j.gie.2024.11.016

[2] Lieberman DA Williams JL Holub JL Race, ethnicity, and sex affect risk for polyps greater than 9 mm in average-risk individuals. Gastroenterology 2014;147(2):351–8. 24786894 10.1053/j.gastro.2014.04.037 PMC4121117

[3] Jacobson BC Anderson JC Burke CA Optimizing bowel preparation quality for colonoscopy: Consensus recommendations by the US Multi-Society Task Force on Colorectal Cancer. Gastrointest Endosc 2025;101(4):702–32. 40047767 10.1016/j.gie.2025.02.010

[4] Hassan C East J Radaelli F Bowel preparation for colonoscopy: European Society of Gastrointestinal Endoscopy (ESGE) Guideline – Update 2019. Endoscopy 2019;51(8):775–94. 31295746 10.1055/a-0959-0505

[5] Makaroff KE Shergill J Lauzon M Patient preferences for colorectal cancer screening tests in light of lowering the screening age to 45 years. Clin Gastroenterol Hepatol 2023;21(2):520–31.e10. 35870766 10.1016/j.cgh.2022.07.012 PMC9852355

[6] United Stated Food and Drug Association. Accessed November 19, 2025. https://www.accessdata.fda.gov/cdrh_docs/pdf23/P230009B.pdf

[7] United Stated Food and Drug Association. Accessed November 19, 2025. https://www.accessdata.fda.gov/cdrh_docs/pdf13/p130017b.pdf

[8] United Stated Food and Drug Association. Accessed November 19, 2025. https://www.accessdata.fda.gov/cdrh_docs/pdf23/P230043B.pdf

[9] Kaltenbach T Anderson JC Burke CA Endoscopic removal of colorectal lesions: Recommendations by the US Multi-Society Task Force on Colorectal Cancer. Gastrointest Endosc 2020;91(3):486–519. 32067745 10.1016/j.gie.2020.01.029

